# COMMUNITY-LEVEL SOCIAL CAPITAL AND COGNITIVE FUNCTION AMONG OLDER ADULTS IN FIVE LOW- AND MIDDLE-INCOME COUNTRIES

**DOI:** 10.1093/geroni/igz038.3016

**Published:** 2019-11-08

**Authors:** Nan Jiang, Nan Lu, Bei Wu

**Affiliations:** 1 Columbia University, New York, United States; 2 Renmin University of China, Beijing, Beijing, China (People’s Republic); 3 NYU Rory Meyers College of Nursing, New York, New York, United States

## Abstract

This study aims to investigate which community social capital components are associated with a higher likelihood of cognitive functions across economically and culturally distinctive low- and middle-income countries (LMICs). We used cross-sectional survey data from the World Health Organization’s Study on global AGEing and adult health (SAGE) 2007-2010. Associations between community-level social capital indicators and global cognitive scores were examined using ordinary least squares regressions and random-effects meta-analyses. The pooled analysis and meta-analyses of within-country effects indicated that trust in neighbors and coworkers were positively associated with cognitive functions for all these countries, whereas the significant effect of perceived neighborhood safety was only found in South Africa and China. Community participation approached a null effect only in South Africa. This finding explains how community social capital may contribute to better cognitive function through community environments, heath systems, and availability of public resources.

